# Patient-derived organoid-guided precision therapy for a very young woman with multidrug-resistant metastatic breast cancer: a case report

**DOI:** 10.3389/fonc.2025.1630761

**Published:** 2025-10-06

**Authors:** Xiaonuo Zhang, Zhen Zhang, Yidan Chen, Yiping Xu, Mingjiao Sun

**Affiliations:** ^1^ Department of Medical Oncology, Hangzhou Cancer Hospital, Affiliated Hangzhou First People's Hospital, West Lake University, Hangzhou, China; ^2^ Hangzhou Cancer Institute, Hangzhou Cancer Hospital, Affiliated Hangzhou First People's Hospital, West Lake University, Hangzhou, China; ^3^ Pathology Department, Hangzhou Cancer Hospital, Affiliated Hangzhou First People's Hospital, West Lake University, Hangzhou, China

**Keywords:** breast cancer, young women, patient-derived organoid (PDO), malignant pleural effusion, drug sensitivity testing, individualized therapy

## Abstract

We reported a case of a 31-year-old female with therapy-resistant, refractory, and metastatic luminal B breast cancer. Using organoids derived from the patient’s malignant pleural effusion for drug screening, we found that the combination of gemcitabine and cisplatin was the most sensitive, considering both IC50 and AUC values. In clinical practice, it was observed that the patient responded well to the selected treatment regimen, resulting in a significant reduction of pleural effusions, a marked decrease in tumor markers (e.g., CA125), and improved performance status (PS 2→1). The organoid model enabled the rational use of the patient’s metabolic waste. It replicates the complexity of human tumors and facilitates extensive screening of beneficial drugs for patient diseases, particularly those with advanced tumors showing heterogeneity and rapid disease progression. This method swiftly identifies the optimal therapeutic drug regimen, minimizing the risk of drug resistance and trial costs, thereby providing maximum patient benefits

## Introduction

Breast cancer is one of the most common malignant tumors affecting women and poses a significant threat to their health (1). In recent years, there has been a trend toward a younger onset of breast cancer (2). In China, most scholars believe that the age of disease ≤35 years old should be defined as young breast cancer. Young women face a high risk of refractory and metastatic disease, even when diagnosed at an early stage (3). Compared to middle-aged and older breast cancer patients, young breast cancer patients exhibit distinct pathological characteristics (4). These include high histological grade, a high rate of HER-2 overexpression, and a higher expression of the BRCA-1 gene (5). Clinical manifestations include a greater likelihood of postoperative recurrence, a higher rate of axillary lymph node metastasis, and a poorer survival prognosis (6). Given that most young breast cancer patients are at an optimal age for work and childbearing, the impact of diagnosis, treatment, and long-term survival can be profoundly adverse to their physical and mental health, as well as on their families and society at large.

Organoids are cell clusters formed through the three-dimensional culture of stem cell-like cells *in vitro*. They can self-renew and self-assemble, displaying structural and functional characteristics that resemble the source tissues. Organoids can be maintained through long-term subculturing and exhibit stable phenotypic and genetic traits (7). This technology has significantly advanced *in vitro* tissue culture methods in recent years. Organoids hold considerable potential for guiding treatment in various patient groups, including early multidrug candidates, advanced multidrug-resistant patients, and individuals with rare tumors that are difficult to treat (8). Only a small number of samples are required to conduct drug sensitivity tests on organoids, thereby providing clinical guidance for medication choices, achieving individualized precision treatment, reducing drug side effects, and ultimately improving the quality of life for patients (9).

## Case report

**Figure 1:**
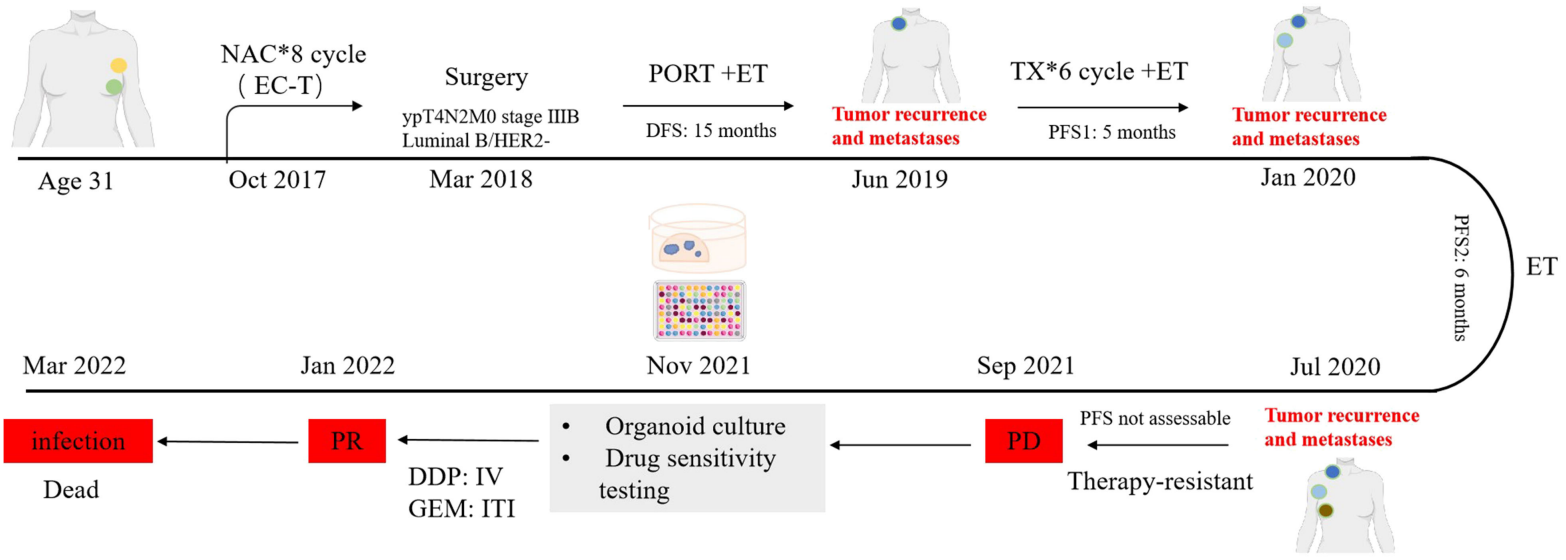
Timeline depicting the clinical history of a patient with breast cancer. NAC, Neoadjuvant Chemotherapy; PORT, Postoperative Radiotherapy; EC-T, Epirubicin + Cyclophosphamide sequential Taxol; ET, Hormonal Therapy; TX, docetaxel + capecitabine; DFS, Disease-free survival; PFS, Progression-free survival; DDP, Cisplatin; GEM, Gemcitabine; IV, Intravenous injection; ITI, Intrathoracic injection. Some elements in this figure were created by FigDraw.

A 31-year-old female without familial cancer history was diagnosed with left breast cancer (ypT4N2M0, stage IIIB Luminal B/HER2−) after eight cycles of neoadjuvant chemotherapy and underwent radical surgery. Adjuvant radiotherapy (left breast and axilla) and endocrine therapy were administered. Disease-free survival (DFS) lasted 15 months until right supraclavicular lymph node metastasis occurred, prompting systemic chemotherapy and endocrine therapy. Progression-free survival (PFS1) was 5 months before progression to the right axillary lymph nodes and left chest wall, leading to a switch in endocrine therapy and ovarian suppression. PFS2 lasted 6 months until contralateral breast metastasis developed. The disease exhibited repeated progression despite multiple lines of chemotherapy (docetaxel, capecitabine, vinorelbine, albumin-bound paclitaxel), with poor response to endocrine therapies. Due to worsening Malignant pleural effusions and multidrug resistance, tumor organoid drug sensitivity testing was pursued. Malignant pleural effusions were collected for organoid culture (Nov 2021- Feb 2022). High-throughput drug screening identified cisplatin and gemcitabine as effective agents. Bilateral intrapleural cisplatin infusion was administered, followed by intravenous gemcitabine on December 10. Post-treatment CT imaging showed a significant reduction of pleural effusions, a marked decrease in tumor markers (e.g., CA125), improved performance status (PS 2→1), and partial response (PR) according to RECIST criteria(Response Evaluation Criteria in Solid Tumors). And other metastatic sites remain stable. Later, due to a secondary infection from the chest wall tumor, there was a persistent fever despite anti-infection treatments while waiting for anti-tumor treatment opportunities. On March 26, 2022, the patient experienced sudden respiratory and cardiac arrest, leading to death. A timeline of the case is presented in **Figure 1**.

## Materials and methods

### Organoid culture

1000 mL of breast cancer pleural effusion was collected, centrifuged, and washed with DPBS (Dulbecco’s Phosphate-Buffered Saline). Red blood cells were lysed with lysis solution (Solarbio, Beijing, China) and terminated with DPBS. Cell pellets were resuspended in Matrigel (Corning, New York, USA) and seeded into a 24-well plate at 40 µL (10000 cells) per well. The concentration of Matrigel in the droplets is 55%. The plate was incubated at 37 °C for 30 minutes to solidify the gel, then 500 µl of MasterAimTM Breast Cancer Organoid Complete Medium (AimingMed, Hangzhou, China) was added. Medium was changed every 3 days, and organoids were passaged every 2 weeks. When organoids reached over 200 µm in diameter and over 80% density, they were treated with TrypLE Express (Invitrogen, Carlsbad, CA, USA), dissociated into single cells, and repassaged, plated, or cryopreserved as needed.

### Histological analysis and immunohistochemistry

200mL breast cancer pleural effusion was collected, centrifuged, and fixed with 10% neutral buffered formalin for 6-24h. Dehydration was carried out using different concentrations of ethanol, followed by clearing with xylene and then infiltration with paraffin for embedding and sectioning. Organoids were fixed with 4% paraformaldehyde overnight, then resuspended in 3% agarose, paraffin-embedded, and sectioned (4µm). The paraffin sections were stained with H&E for histological analysis after deparaffinization. Immunohistochemistry analyses were performed following a two-step method. The sections were incubated for ER, PR, HER2, and Ki67 antibodies(Cell Signaling Technology, Boston, USA) after antigen retrieval and blocking. After secondary antibodies(Absin, Shanghai, China) incubation, the staining was visualized by DAB substrate and counterstained with hematoxylin.

### Whole exome sequencing and data analysis

The total DNA was extracted using the QIAGEN DNeasy Blood & Tissue Kit (QIAGEN). Then, the DNA, which was fragmented with the Covaris Focused-ultrasonicator M220 (Covaris), was used for sequencing library construction. Exome capture was performed using the Human Exome 2.0 Plus (Twist Bioscience) following the vendor’s recommended protocol. The final libraries were sequenced with paired-end 150 bp reads on the Illumina NovaSeq 6000 Sequencing System (Illumina) at LC-Bio Technology Co., Ltd (Hangzhou, China).

Before alignment, low-quality reads (such as those containing sequencing adaptors or with nucleotide quality scores below 20) were removed using fastp (10). For the alignment step, Burrows-Wheeler Aligner (BWA) (11) was used to align reads to the reference genome hg19. As the first post-alignment processing step, Picard tools (http://broadinstitute.github.io/picard/) were employed to identify and mark duplicate reads in the BAM file. The second post-alignment step involved performing local read realignment to correct potential errors around indels. Base quality score recalibration was then carried out before variant calling to reduce systematic biases. For somatic variant calling, we used GATK’s Mutect2 tool in tumor-only mode. This mode utilizes a pre-assembled Panel of Normals (PON), built from hundreds to thousands of normal samples, to exclude germline variants (12, 13).

### Drug screening

According to the passaging process described above, the collected single cells were resuspended in an appropriate volume of MasterAimTM Breast Cancer Organoid Complete Medium (AimingMed, Hangzhou, China) containing 5% Matrigel. Using a multichannel electronic pipette, the cell suspension was dispensed into a pre-cooled 384-well white ultra-low attachment plate (Corning, New York, USA) at a volume of 40 μL containing 1000 cells per well. After 48 hours, six concentrations of cisplatin, Anlotinib, Gemcitabine combined with cisplatin, Gemcitabine, Paclitaxel, and a DMSO control were added, with three replicates for each concentration. Four days later, 40 μL of CellTiter-Glo 3D reagent (Promega, Madison, WI, USA) was added to each well. The plate was shaken at room temperature for 30 minutes to lyse the cells. Luminescence was measured using a multimode microplate reader (Molecular Devices). IC50 and AUC values for each drug were analyzed using GraphPad Prism 6.

### Statistical analysis

All data analyses were conducted using GraphPad Prism 6. Quantitative data are expressed as mean ± standard deviation (X ± SD), and comparisons among multiple groups were performed using one-way analysis of variance (one-way ANOVA).

## Results

### Establishment of an advanced recurrent breast cancer patient-derived organoid

**Figure 2:**
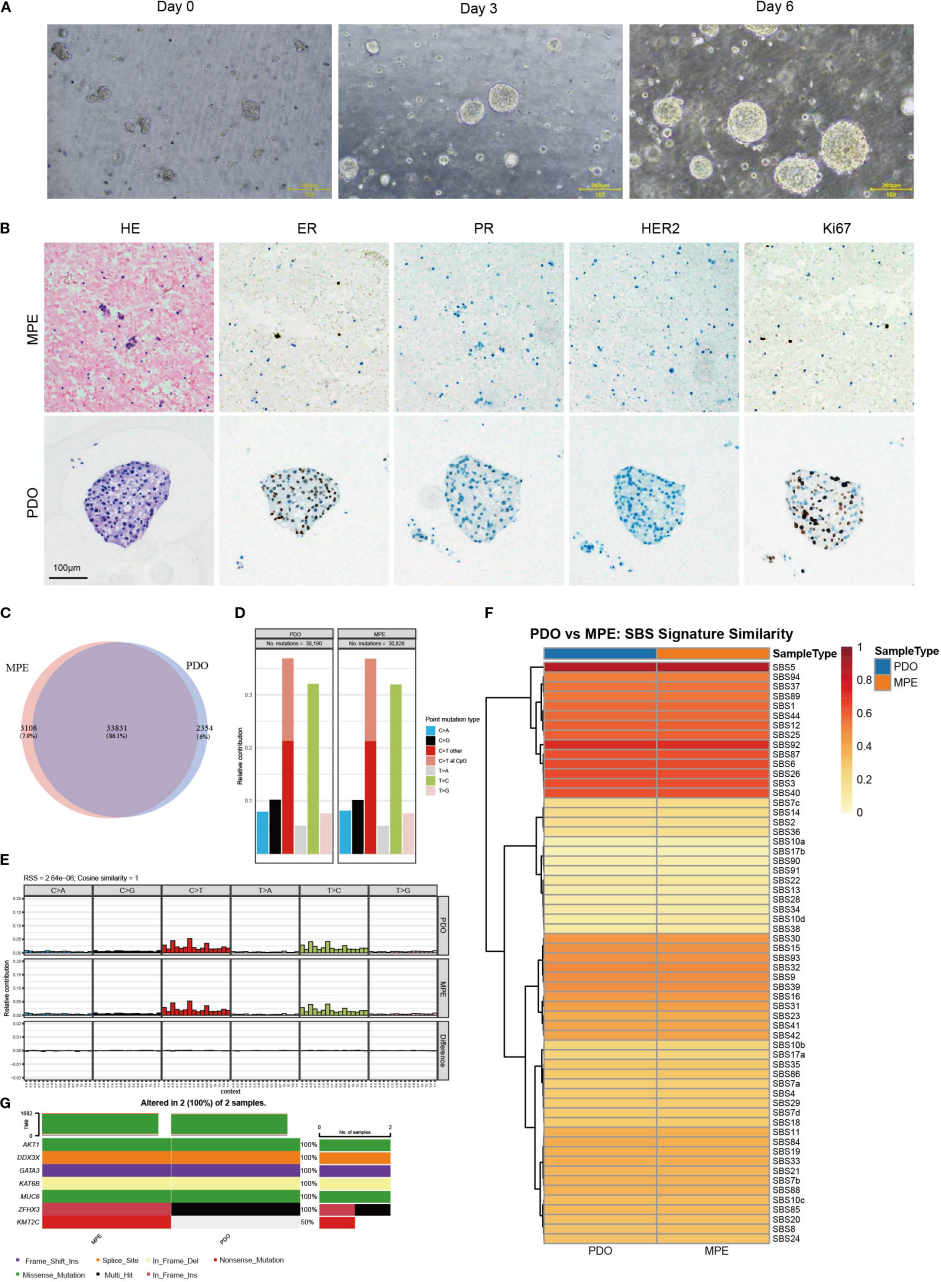
Organoids morphology, pathological characterization, and genetic profiling. **(A)** Morphological images of organoids at different growth stages. Scale bar = 200 μm. **(B)** Representative images of H&E and immunohistochemistry staining of malignant pleural effusion (MPE) and patient-derived organoids (PDO), Scale bar = 100 μm. **(C)** Venn diagram showing somatic mutations in PDO and MPE. **(D)** Bar plots showing the frequency of different point mutation types in PDO and the paired MPE. **(E)** Mutational signature profiles representing 96 nucleotide combinations in PDO and MPE samples. **(F)** Heatmap comparing the COSMIC mutational signatures in PDO and MPE. **(G)** Waterfall chart illustrating the mutation types of breast cancer-related genes reported from the TCGA database for PDO and MPE samples.

To establish the organoid model, we centrifuged the turbid pleural effusion and used a red blood cell lysis buffer to remove red blood cells from the precipitate. The treated cell precipitate was suspended in Matrigel^®^ to simulate the tumor extracellular matrix (ECM) environment. Under the microscope, the newly inoculated cells included individual cells and cell clusters (approximately 50 μm in diameter). After three days of culture at 37 °C with 5% CO_2_, the organoids entered the logarithmic growth phase, increasing rapidly in number and size. By day six, many organoids had grown, with the largest diameter reaching about 200 μm (**Figure 2A**), indicating the successful establishment of organoid cultures from late-stage recurrent breast cancer biopsy samples.

### The pathologic features and genetic characteristics of breast cancer organoids derived from pleural effusion were consistent with the original pleural effusion

We conducted hematoxylin and eosin H&E staining on patient-derived organoid(PDO) and tumor cell sections from parental malignant pleural effusion(MPE) (**Figure 2B**), revealing that the organoids’ phenotypic characteristics closely matched the original pleural effusion’s histological features. The tumor cells showed significant cellular and nuclear pleomorphism, with variable cell sizes, enlarged nuclei, disordered arrangement, and abnormal structure, typical of malignant cells. Immunohistochemical staining also showed that the organoids retained key breast cancer biomarker expression patterns consistent with the original pleural effusion: ER, PR, and HER2, and positive for Ki-67. These biomarkers are crucial for predicting treatment response and prognosis. In summary, pleural effusion-derived tumor organoids closely match the original pleural effusion in both histopathological features and key biomarker expression, making them a high-fidelity model for studying breast cancer behavior and evaluating therapies.

WES was conducted in PDO and MPE to assess whether the cultured organoids retain the parental genomic features. After filtering somatic sites using GATK’s FilterMutectCalls, we performed a concordance analysis of the somatic mutations in both samples, revealing an 86.1% concordance rate for shared mutations (**Figure 2C**). Additionally, we noted that the relative contributions of point mutations (**Figure 2D**), mutation characteristic profiles of 96 nucleotide combinations (**Figure 2E**), and COSMIC mutational signatures (**Figure 2F**) were well-preserved in the PDO and MPE. Further analysis showed a significant number of concordant mutations within breast cancer-related genes from the TCGA database across both samples (**Figure 2G**). These findings underscore the remarkable ability of pleural effusion-derived tumor organoids to maintain the genetic architecture of primary tumors, making them highly reliable models for breast cancer drug susceptibility testing.

### Drug sensitivity test for organoid recurrent breast cancer as a personalized treatment tool

**Figure 3:**
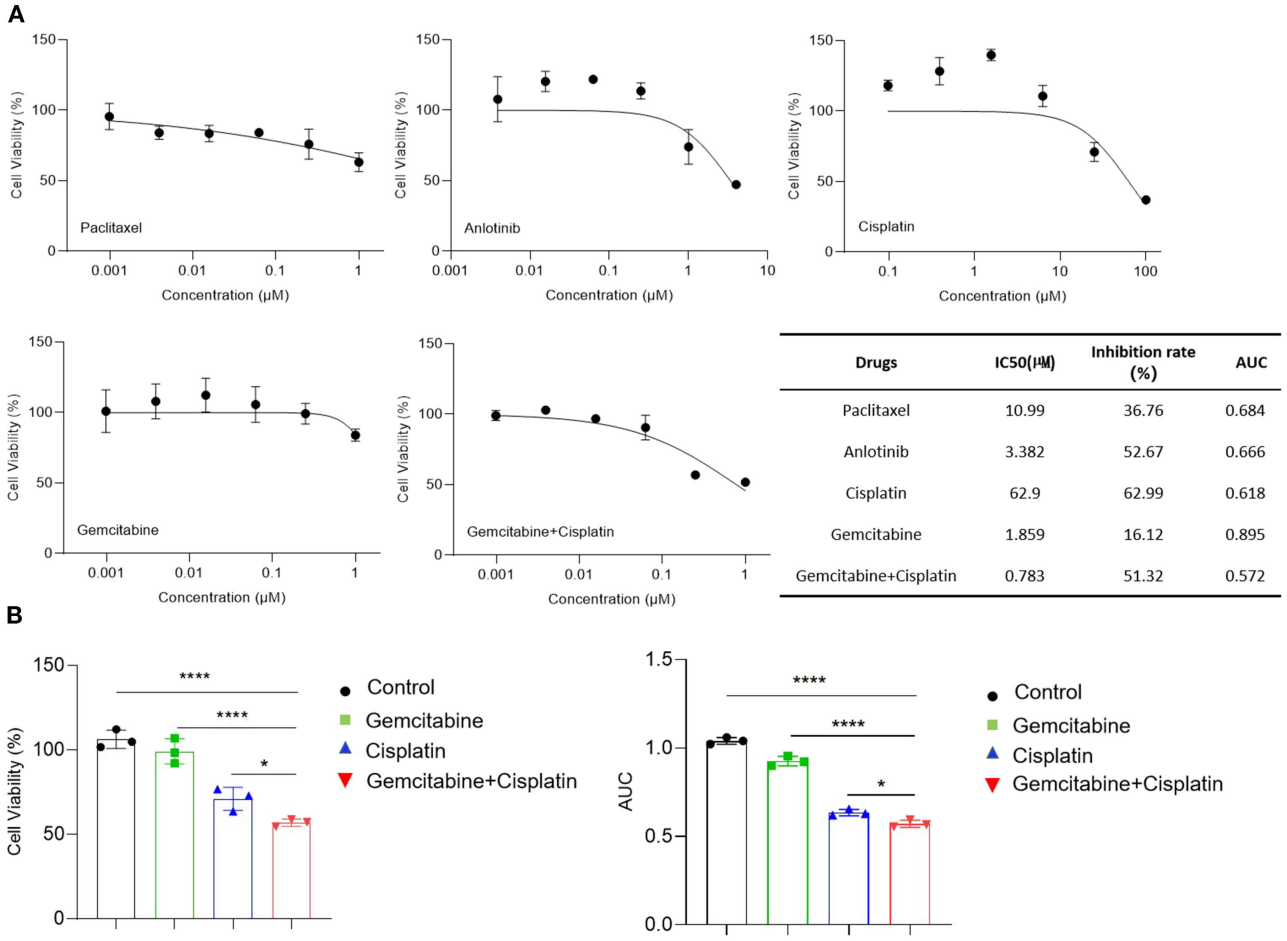
Drug sensitivity of tumor organoids. **(A)** Dose -response curves for tumor organoids treated with Paclitaxel, Anlotinib, Cisplatin, Gemcitabine, and Gemcitabine + Cisplatin, along with the IC50, inhibition rate, and AUC for each drug in the table. Inhibition rate refers to the inhibition at the maximum concentration of each drug. **(B)** Statistical analysis of cell viability and AUC of organoids treated with cisplatin, gemcitabine, and gemcitabine + cisplatin. *p<0.05; ****p<0.0001. Results are shown as mean ± SD from three replicate wells for each drug.

We performed a drug screening experiment on breast cancer organoids. Five drug or combination therapies were tested. The results showed that the combination of gemcitabine and cisplatin had favorable antitumor effects *in vitro*. The cytotoxic response curves and IC50 values of the organoids to each drug are shown in **Figure 3A**. The IC50 values of anlotinib, cisplatin, and the gemcitabine plus cisplatin regimen were within the tested range, with maximum inhibition rates below 50%. According to literature reports, there is a strong correlation between IC50 and AUC in organoid drug sensitivity assays (14). Therefore, the AUC values were also analyzed, and the cisplatin plus gemcitabine regimen had the smallest AUC. Additionally, the effects of the combination regimen on cell viability and the corresponding AUC values show significant differences compared to those of the monotherapy regimens (**Figure 3B**). Considering both IC50 and AUC, the organoid drug sensitivity test indicated that the gemcitabine plus cisplatin regimen was the most sensitive.

**Figure 4:**
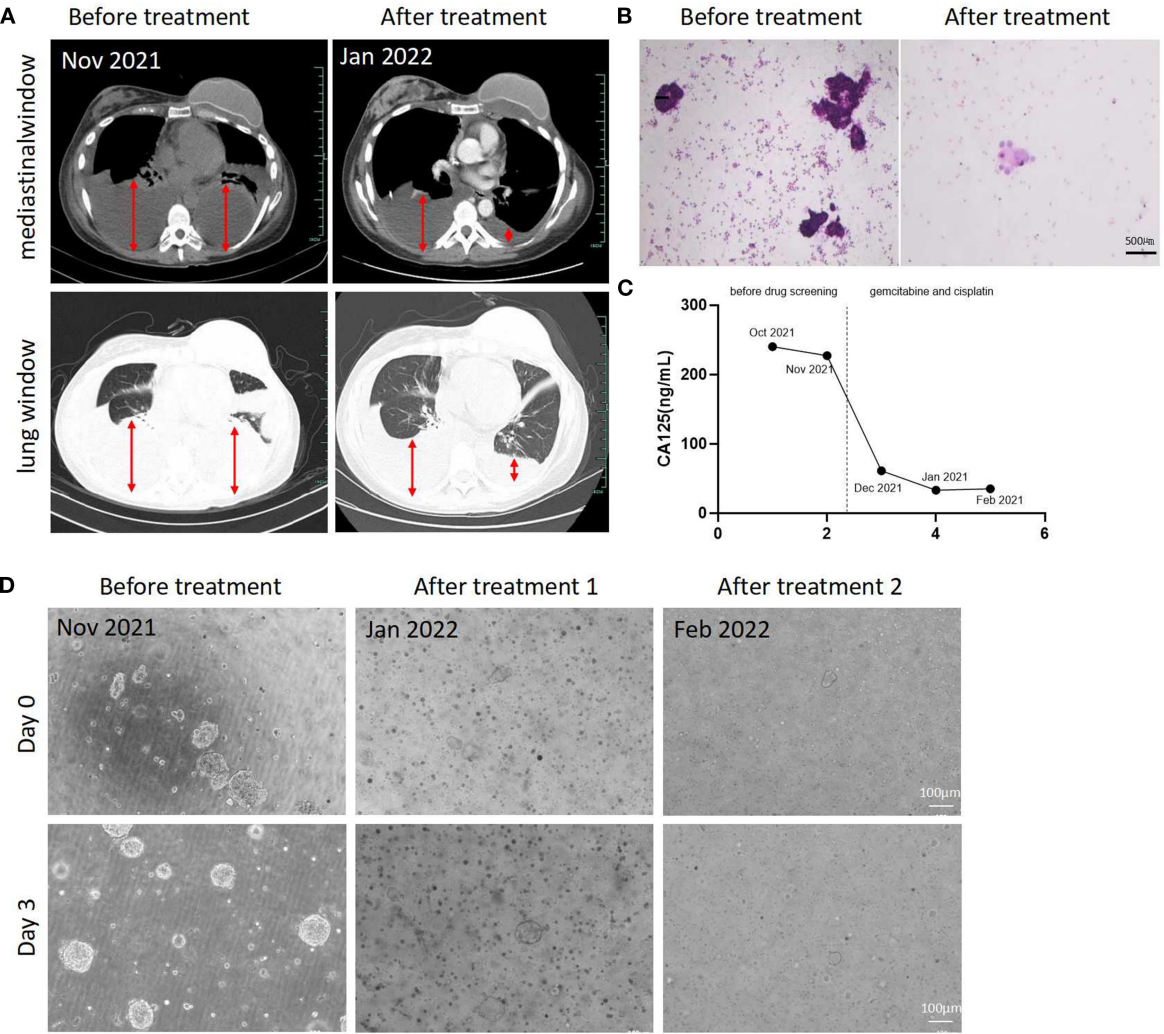
Evaluation of the effectiveness of drug guided by Organoid drug sensitivity tests. **(A)** CT images of this patient before and after Gemcitabine and Cisplatin treatment. **(B)** Cytological pathological images of thoracic pleural effusion before and after the initiation of Gemcitabine and Cisplatin therapy. Scale bar = 500 μm. **(C)** Curve of CA125 level changes in peripheral blood. **(D)** Images of organoid growth before and after Gemcitabine and Cisplatin treatment. Scale bar = 100 μm.

Based on the drug sensitivity test, we recommended the gemcitabine and cisplatin combination therapy for the patient. After two cycles, the patient showed significant reductions in pleural effusions (**Figure 4A**), tumor cells in the effusion (**Figure 4B**), and tumor markers (e.g., CA125) (**Figure 4C**), improved performance status (PS 2→1), and partial response (PR) by RECIST criteria. We collected pleural effusion for organoid culture. Microscopic observations revealed that the cell clusters in the second culture were smaller and fewer than the first culture at day 0. After 3 days, some organoids grew to about 100 micrometers, but their number was still lower than in the first culture, with many black cells observed, possibly indicating drug-induced apoptosis or necrosis.

After four cycles, the patient was re-evaluated. Tumor marker levels remained significantly decreased, and we recollected the pleural effusion for organoid culture again. The cell clusters were smaller and fewer than in the second culture (**Figure 4D**). These results show that the gemcitabine and cisplatin regimen was effective both *in vitro* and in the patient initially. Using organoid models for drug screening can provide personalized treatment for recurrent breast cancer patients, improving outcomes and reducing unnecessary side effects.

## Discussion

Compared with Western countries, China has a higher proportion of young women with breast cancer (15). The invasive, rapid development and poor prognosis of young female breast cancer are related to its unique pathological features (16). Of the four molecular subtypes of breast cancer, the aggressive ones are more common in younger women. At the same time, Young female breast cancer patients are often in their peak fertility period, characterized by strong ovarian function and high estrogen levels. However, these patients tend to have lower compliance with hormone therapy. The elevated estrogen levels can contribute to a higher degree of tumor malignancy, early metastasis, and poor prognosis.

This case involves a 31-year-old woman diagnosed with breast cancer who had axillary lymph node metastasis at the time the tumor was discovered. The surgery was performed in the late stages of her illness. Postoperative pathology revealed the presence of a vascular cancer thrombus, involvement of the papillary dermis, and metastasis to the axillary lymph nodes. These findings indicate a high risk of relapse. Radiotherapy, endocrine therapy, and other classical systemic treatments were given after surgery. DFS lasts only 15 months. Compared with older women, younger women had less benefit from adjuvant endocrine therapy and higher resistance to tamoxifen (17). At the same time, young patients are more likely to develop chemotherapy resistance, which may be related to tumor stem cell enrichment or DNA repair defects (16). The rapid progression of the disease, along with resistance to endocrine and chemotherapy treatments, makes follow-up treatment crucial for patients. Additionally, the physical condition of the patients and the severe side effects of the drugs indicate that we cannot afford to experiment with different treatments. At this critical juncture, we need a model that can quickly simulate the tumor status of patients and identify suitable drug substitutions.

In this study, we successfully established tumor organoid models derived from the malignant pleural effusion of patients. We identified a synergistic therapeutic regimen of gemcitabine and cisplatin through these models. The efficacy of this regimen was further validated in clinical practice, demonstrating significant therapeutic effects. This finding provides important insights for the personalized treatment of advanced cancer patients.

Organoids hold significant value for clinical decision-making in refractory tumors. Traditional chemotherapy regimens are often selected based on evidence-based medicine guidelines, which may lag in the context of highly heterogeneous and rapidly progressing advanced tumors. In this study, we innovatively utilized malignant pleural effusion, an easily accessible “metabolic waste product,” to construct organoid models. Compared with traditional tumor tissue biopsies, liquid samples are easier to obtain and less invasive, especially suitable for advanced patients who cannot undergo repeated biopsies. Moreover, malignant pleural effusion is rich in tumor cells and components of the tumor microenvironment (such as immune cells and fibroblasts) (18), preserving the heterogeneity and microenvironmental characteristics of the primary tumor (19).

Traditional 2D cell lines lose tumor cell heterogeneity and tissue-of-origin features during long-term culture, failing to simulate the primary tumor’s 3D environment, tissue functions, and signaling pathways. In contrast, organoids from malignant pleural effusion better simulate *in vivo* drug responses due to their preserved heterogeneity and treatment sensitivity. They can be rapidly cultured into micro-tumors for high-throughput screening, reducing the “trial-and-error” period. In this case, organoid culture and regimen determination took only 2–3 weeks, faster than NGS-guided targeted therapy.

In this case, we monitored both IC50 and AUC to avoid the risk of false positives associated with single-parameter assessments. Notably, the rapid decline in CA125 levels in the patient coincided with a significant reduction in malignant pleural effusion and improvement in physical status, confirming the model’s reliability in predicting clinical efficacy. Additionally, the successful establishment of an organoid model from malignant pleural effusion in a patient with triple-negative breast cancer was also validated.

Based on the drug sensitivity results, cisplatin intrathoracic injection combined with gemcitabine intravenous chemotherapy was adopted in clinical practice. On the one hand, after multiple lines of treatment, the patient had severe bone marrow suppression, and a large amount of pleural effusion led to poor physical condition. Monotherapy via intravenous administration was chosen for its higher safety profile. On the other hand, cisplatin intrathoracic perfusion chemotherapy is a local treatment that allows the drug to directly act on the metastatic lesions, rapidly and effectively controlling the pleural effusion while avoiding the toxic and adverse reactions associated with systemic therapy.

Despite the promising outcomes of this study, organoids still have significant limitations. The success rate of organoid culture is highly dependent on the quality of the samples. The activity of tumor cells in pleural effusion exceeds 40%, which ensures the success rate of organoid culture. Moreover, organoids lack critical components of the tumor microenvironment, such as neural tissue, blood vessels, immune cells, and microbiota, which play important roles in studying tumor evolution, drug resistance mechanisms, and new drug development. In the future, co-culture systems incorporating patient-derived immune cells should be explored to enhance clinical relevance.

## Data Availability

The data presented in the study are deposited in the GSA repository, accession number HRA013442.
